# Challenges and Opportunities for Adult Vaccine Coverage: Insights for Healthcare Professionals Focusing on Herpes Zoster in Mexico

**DOI:** 10.3390/vaccines12121441

**Published:** 2024-12-21

**Authors:** María Yolanda Cervantes-Apolinar, Adriana Guzman-Holst, Abiel Mascareñas De los Santos, Alejandro Ernesto Macías Hernández, Álvaro Cabrera, Argelia Lara-Solares, Carlos Abud Mendoza, Daniel Motola Kuba, Diana Fabiola Flores Díaz, Fernanda Salgado Gomez, Graciela Elia Castro-Narro, Javier Nieto, José Antonio Mata-Marín, José Fernando Barba Gómez, Juan Carlos Tinoco, Juan Manuel Calleja Castillo, Maria Margarita Contreras Serratos, Nathali Castellanos Ramos, Oscar Rosas Carrasco, Raúl Ricaño, Gloria C. Huerta García

**Affiliations:** 1 GSK México, Torre Mitikah Piso 19 y 20, Circuito Interior Avenida Río Churubusco 601, Col. Xoco. Alc. Benito Juárez, Mexico City 03330, Mexicojnietoguevara@gmail.com (J.N.);; 2 University Hospital Monterrey, Monterrey 64460, Mexico; 3 Microbiology and Infectious Diseases, Department of Medicine and Nutrition, University of Guanajuato, Guanajuato 36000, Mexico; aaeemmhh@yahoo.com; 4 Ixtapaluca High Specialty Regional Hospital, Ixtapaluca 56530, Mexico; 5 National Institute of Nutrition “Salvador Zubirán”, Mexico City 14080, Mexico; 6 Hospital Central y Facultad de Medicina de la Universidad Autónoma de San Luis Potosí, San Luis Potosí 78290, Mexico; 7 Médica Sur Hospital, Mexico City 14050, Mexico; 8 National Cancer Institute, Mexico City 14080, Mexico; 9 Bavarian Nordic AG, 6301 Zug, Switzerland; 10 Latin-American Association for the Study of the Liver (ALEH), Santiago de Chile 7591047, Chile; 11 National Medical Center La Raza, Mexico City 02990, Mexico; 12 Dermatologic Center of Jalisco, Guadalajara 45190, Mexico; 13 General Hospital of Durango, Durango 34234, Mexico; 14 ABC Medical Center, Mexico City 05348, Mexico; 15 National Medical Center Siglo XXI, Mexico City 06720, Mexico; 16 Ibero Geriatric Clinical and Research Center, American University, Mexico City 06700, Mexico

**Keywords:** vaccines, herpes zoster, healthy aging, immunocompromised, Mexico

## Abstract

Herpes zoster (HZ) is a common disease in older adults and immunocompromised patients, and is frequently associated with long-term complications that impact quality of life. Fortunately, more than one vaccine against HZ is now available in Mexico. Two expert consensus groups discussed adult vaccination strategies in Mexico, focusing on HZ in older adults and immunocompromised individuals; their insights are reported here. HZ is usually treated inappropriately in Mexico. Late diagnosis and suboptimal management are common, as is a lack of treatment options, particularly for pain, which is often unresponsive to standard painkillers. Improving vaccination rates against HZ in Mexico is therefore important, but several barriers to HZ vaccination exist. It is not included in the national vaccination schedule, where included vaccines usually have higher coverage. Actions to overcome barriers include improving awareness of HZ and vaccine availability, developing and promoting guidelines and recommendations for vaccination, and expanding access and infrastructure for vaccination.

## 1. Introduction

Herpes zoster (HZ), commonly known as shingles, is characterized by a painful, blistering rash, and is frequently associated with complications, including post-herpetic neuralgia (PHN; debilitating pain that persists after resolution of the rash), secondary bacterial infection, eye involvement, neurological complications, vasculopathy, and disseminated infections [1]. The disease is caused by reactivation of the varicella-zoster virus following an episode of chickenpox, after which the virus becomes latent in the dorsal root and cranial nerve ganglia [2].

HZ is a common disease whose incidence rises with age, increasing sharply after 50 years of age [2]. The global incidence of HZ in individuals ≥50 years of age has been estimated to be between 5 and 11 cases per 1000 person-years [3]. Its incidence is higher in immunocompromised individuals than in immunocompetent individuals [2]. It is associated with a considerable economic burden due to the costs of treating the disease and its complications and due to lost productivity among working-age people [4,5,6].

Two vaccines against HZ are currently available: the live attenuated zoster vaccine (zoster vaccine live [ZVL], *Zostavax*, MSD) and the recombinant zoster vaccine (RZV, *Shingrix*, GSK). ZVL contains live attenuated varicella-zoster virus, while RZV consists of a recombinant subunit varicella-zoster virus glycoprotein E and the AS01_B_ adjuvant system. In phase 3 clinical trials, both vaccines have been shown to reduce the risk of developing HZ in older adults [7,8,9]; however, the vaccine efficacy of RZV is higher than that of ZVL in all age groups and RZV can also be used in immunocompromised adults. The US Advisory Committee on Immunization Practices has made a preferential recommendation in favor of RZV [10]. Both vaccines are registered in numerous countries worldwide, including Mexico. Currently, RZV is the only HZ vaccine available on the market in Mexico, but it is not included in the National Immunization Program (NIP).

The coronavirus disease 19 (COVID-19) pandemic has raised worldwide awareness of the importance of vaccination and has been a catalyst for adult vaccination. Therefore, this is a good moment to focus efforts on expanding vaccination among adults, particularly older adults who are at higher risk of morbidity and mortality from infectious diseases. Adult vaccination coverage rates are suboptimal in many countries [11]. For example, in the US, influenza vaccine coverage in 2017–2018 was 48% in adults ≥50 years of age and 72% in those ≥65 years of age [12]. In Europe, the median coverage rate of influenza vaccine in older adults was 47% in 2016–2017 [13]. Sociodemographic, health-related, and attitudinal factors all influence vaccine uptake in older adults [11]. Vaccine hesitancy remains a problem worldwide, and the World Health Organization has described vaccine hesitancy as one of the greatest threats to global health [14]. Many factors influencing vaccine hesitancy are related to communication, underlining the importance of effective communication in enhancing vaccination coverage [15]. Optimizing vaccine information, effective use of online channels, and education and awareness campaigns for both healthcare providers and the general public are believed to be important for promoting vaccination among older adults [11,15].

In this article, we report the insights and opinions of two expert consensus groups convened to discuss adult vaccination strategies in Mexico, focusing on HZ in older adults and immunocompromised individuals. The article focuses on the discussions that took place during the expert panels and does not attempt to give a comprehensive overview of all issues affecting adult vaccination in Mexico.

**Figure 1:**
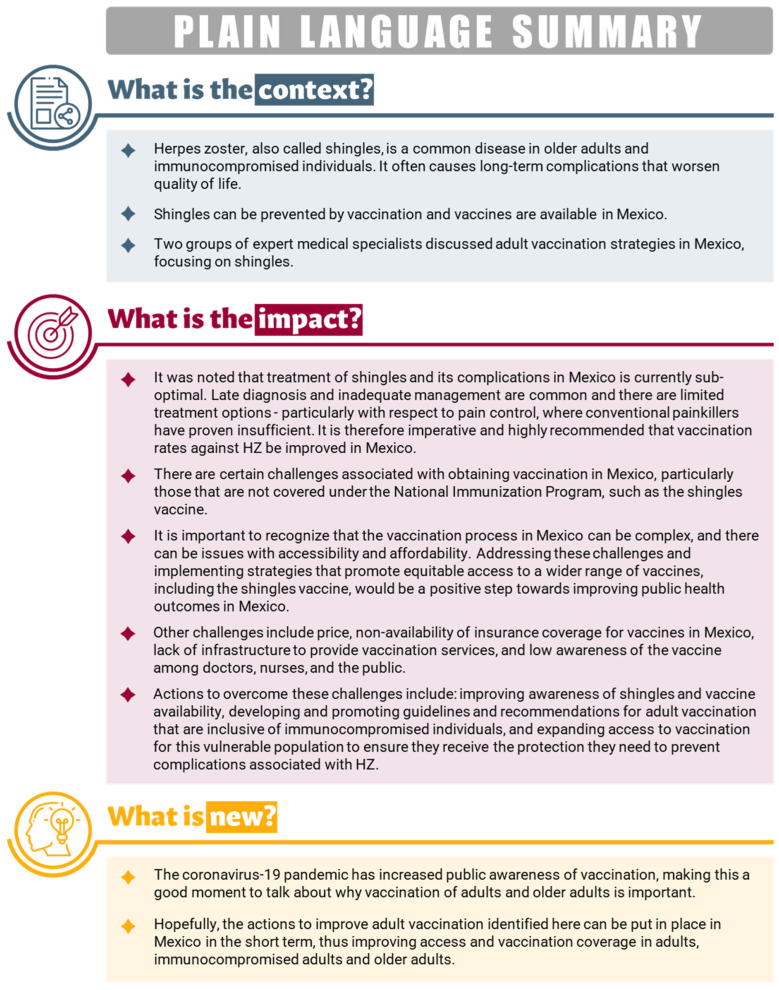
Plain language summary.

Figure 1 provides a plain language summary that explains the relevance, results, and impact of the discussion between two groups of expert medical specialists.

## 2. Current Status of Adult Vaccination in Mexico

It is important to note that, while the First Mexican Consensus on Adult Vaccination in 2017 recommended a comprehensive list of vaccines, including vaccination against HZ [16], not all of the vaccines recommended by the consensus group are currently included in the NIP. In addition, vaccine coverage is limited, and only 44% of adults aged 60–64 years and 31% of those aged >65 years have complete vaccine coverage as recommended by the NIP [17]. Analysis of data from the 2012 National Health and Nutrition Survey (Encuesta Nacional de Salud y Nutrición, ENSANUT) showed that vaccine coverage in adults aged 60–64 years was 46.5% for the complete scheme (tetanus and diphtheria [Td] and influenza), 66.2% for Td, and 56.0% for influenza vaccines [18]. For adults aged ≥65 years, coverage was 44.0%, 69.0%, 63.3%, and 62.0% for the complete scheme (Td, pneumococcus, and influenza), Td, influenza, and pneumococcal vaccines, respectively [18]. Updated ENSANUT data reported lower rates of vaccination coverage among adults aged ≥65 years in 2021 compared with 2012, with rates of 24.8%, 61.2%, 55.6%, and 36.3% for the complete scheme, Td, influenza, and pneumococcal vaccines, respectively, in 2021 [19]. Vaccination coverage was increased among persons using preventative health services, those with morbidities, beneficiaries of any health system institution, and those who had a National Health Card [19]. There was no clear relationship between vaccine uptake and other factors such as sex, marital status, speaking an indigenous language, literacy, education, employment, depression, or residing in an urban environment [19]. Another study found that national vaccination coverage in Mexico in adults ≥60 years of age was 56.5% for influenza, 44.3% for pneumococcal disease, and 61.8% for tetanus; almost 20% of adults aged ≥60 years had no vaccine coverage at all [20].

## 3. Why Is It Important to Vaccinate Against HZ in Mexico?

HZ results in a considerable health burden in Mexico. A systematic review and meta-analysis which characterized the epidemiology and burden of HZ in Latin America and the Caribbean reported the incidence of HZ in higher-risk populations, describing a cumulative incidence ranging from 318 to 3423 cases of HZ per 100,000 persons per year of follow-up [21]. The incidence density ranged from 6.4 to 36.5 cases per 1000 person-years. Age was identified as a major risk factor for HZ incidence, which increased significantly in persons >50 years of age [21]. Immunocompromised individuals were at higher risk of experiencing HZ complications than immunocompetent individuals [21]. A large cross-sectional study in Mexico reported overall seroprevalence of varicella-zoster virus of 85.8% in individuals aged 1–70 years [22]. An analysis of the surveillance database of the Mexico Ministry of Health (Secretaría de Salubridad y Asistencia/Dirección General de Información en Salud) reported 53,030 emergency room visits due to HZ in Mexico between 2011 and 2020, corresponding to a cumulative incidence rate of 1.04, 1.47, and 1.91 cases per 1000 adults aged ≥50, ≥65, and ≥80 years, respectively [23]. It is important to note that these values are likely to be underestimated due to the fact that they do not capture individuals who did not seek care at an emergency service, or who sought care from private healthcare providers or pharmacy physicians [23]. Furthermore, HZ is a non-notifiable disease in Mexico and is probably under-reported [21,23]. The same analysis of Ministry of Health data reported 4172 hospitalizations due to HZ (2011–2020), of which 59% occurred in adults ≥50 years, and 263 deaths (2011–2019), of which 81% occurred in adults ≥65 years [23].

Healthcare resource use and costs (direct and indirect) associated with HZ in Mexico are considerable. The Mexican Institute of Social Security reported an average of 443 annual hospitalizations 2010–2017, with an average stay of 4.8 days, while the Secretaría de Salud reported an average of 131 annual hospitalizations 2018–2019, with an average stay of 3.8 days [24]. Total direct and indirect costs per HZ case in 2015 were estimated at USD 681 with no PHN, rising to USD 752 with PHN (based on 1 Mexican peso equal to USD 0.06) [25].

## 4. Insights from the Expert Panels

### 4.1. Patient Journey

Despite the significant burden of HZ and its impact on quality of life, the condition is often not well treated, and the associated risk is misperceived. Both the public and healthcare practitioners, including primary care practitioners and specialists who do not usually treat complications of HZ, are often not adequately informed about the risks associated with HZ, leading to misdiagnosis and mismanagement of the condition. Primary care practitioners, in particular, might not recognize symptoms occurring within the first 72 h of the appearance of the rash, or might confuse a rash around the mouth or genitalia with herpes. This can lead to delays or failures to diagnose. Additionally, when HZ is diagnosed, there might be issues with adequate management, including the use of inappropriate topical medications, inadequate doses of antivirals, and poor choice of analgesia. These challenges highlight the need for improved education and awareness among both the public and healthcare providers in Mexico to ensure timely and effective management of this condition.

Patients often experience very late referrals when it comes to specialized care, which is a common issue in many countries, including Mexico. Therefore, it is important to improve primary care management of patients with HZ to ensure timely treatment with appropriate drugs at the correct dose. If the disease occurs in patients with comorbidities or if patients develop complications, prompt referral to specialists such as geriatricians, neurologists, and dermatologists must be warranted. Improving primary care management, timely treatment, and prompt referral can prevent worsening of symptoms, reduce the risk of complications, and improve outcomes for patients with HZ.

One of the most significant challenges in Mexico is the lack of options to treat PHN in patients ≥60 years of age. Many drugs recommended by international guidelines are not appropriate for older patients because of drug–drug interactions in patients receiving multiple medications or because they might have greater effects on the nervous system in older patients. Few physicians other than geriatricians know that dose adjustment is required according to factors such as renal clearance, frailty criteria and drug–drug interactions. Additionally, there is a danger that PHN might become drug-resistant. Another issue is the absence of highly specialized pain management required for patients with HZ in public institutions in Mexico, and the limited availability of such care even in private institutions where it comes at a high cost.

### 4.2. Barriers to Vaccinating Against HZ in Mexico

Considering all the factors described above, prevention of HZ via vaccination is of critical importance in Mexico. However, many barriers exist. The general public and healthcare professionals (HCPs) lack awareness of the risk of HZ, including its high incidence and the severity of its symptoms and long-term consequences. Thus, the need for prevention is not well understood. In addition, there is a lack of awareness of the importance of adult vaccination, with the greatest focus being on childhood vaccination. Many HCPs do not know how to obtain vaccines for adults outside of the NIP; there is a lack of infrastructure for adult vaccination; and it is poorly covered both in undergraduate medical schools and later, during postgraduate training. This means that many HCPs do not consider recommending vaccinations for their adult patients or do not know which patients are eligible for which vaccines. Many HCPs do not realize that HZ vaccines are available.

Medical societies are important in Mexico, as they set the standard of care and are responsible for disseminating information for HCPs in their specialty area. However, the different specialties focus on treatment guidelines and recommendations, and not all include vaccination as a general preventive measure, particularly if the vaccine does not directly prevent the disease or its complications. This can lead to a lack of awareness about adult vaccination among specialists. Furthermore, current treatment protocols for immunocompromised patients do not fully incorporate vaccination as a preventive measure. Additionally, the specialists treating these patients generally do not provide vaccination services.

One of the key issues related to HZ vaccination is the lack of inclusion in the NIP. By incorporating the vaccine into the program, healthcare providers and the general population can become more aware of its importance. Without sufficient information from health authorities, the public and providers might not be familiar with the HZ vaccine nor recognize the benefits it offers. When recommendations for vaccination come from health authorities, there is a greater likelihood of stimulating demand for the vaccine, which can increase its availability. Additionally, inclusion in the national program can improve access to and increase coverage of the HZ vaccine.

Other barriers to HZ vaccination are difficulties related to access, price, and insurance coverage. In Mexico, private insurance does not cover preventive care. There is often limited local access to vaccines that are not included in the NIP. Many HCPs who treat adults do not have access to vaccination centers and do not know where to refer patients for vaccination. Currently, there is little incentive for HCPs who treat adults to invest in the infrastructure and training required to provide vaccination services.

### 4.3. Steps to Improve HZ Vaccination Rates in Mexico

Three key areas are important to consider when it comes to increasing HZ vaccination coverage in Mexico: (1) improving awareness of HZ and vaccine accessibility; (2) developing and promoting guidelines and recommendations; (3) improving access to vaccination.

Disease and vaccination awareness campaigns for the public are crucial to encourage public demand for vaccination, with clear messages about the severity of disease and importance of vaccination. An omnichannel approach is needed. In addition to traditional patient information material distributed by HCPs, other pathways such as social media, celebrities and influencers, and television (e.g., soap operas, interviews with experts) can be used to portray the experience of HZ disease accurately and emphasize the importance of vaccination. It is also important to work with patient associations, including organizations for immunocompromised patients. Individuals with personal experience of HZ (personal illness, illness of family or friends, or participation in a clinical trial) can act as ambassadors to disseminate information and raise awareness through testimonials. Use of non-medical language to describe HZ, such as “culebrilla, cinturón de la reina/lo orinó un grillo/latigazo del diablo (norte)”, might be helpful. Another useful approach aimed at the general public might be the production of simple and easy-to-understand educational material linking vaccination, nutrition, and personal care to sustain immune fitness throughout life, leading to a better quality of life.

Educational campaigns for HCPs on disease and vaccination awareness are also needed to address the need in HCP training where vaccination for adults is not given priority. It is important to work with medical societies and academies to achieve multidisciplinary consensus, disseminate information, and encourage HCPs to recommend vaccination. Regular continuation of medical education is likely to be an effective communication channel, with the most favored channels being medical and hospital societies, colleges, associations, and the pharmaceutical industry. Congresses and regular sessions with societies that grant certificate endorsement provide the opportunity for presentation of clinical cases, emphasizing the impact of HZ on quality of life, as well as the importance of adult vaccination, healthy aging, and immune fitness. Academic sessions for students, residents, and nurses should also play a role. Finally, HCPs should be encouraged to participate in clinical trials as a way of updating knowledge and gaining new experience.

Local guidelines and recommendations are key to improving vaccination rates and it is important to create a culture of preventing disease through vaccination among both the public and HCPs. The role of the HCP, particularly the primary care physician, is key, as they should be recommending vaccination to their patients and vaccination recommendations should be part of routine adult check-ups. However, it has been observed that a significant number of HCPs are not fully informed about the Mexican consensus on adult vaccination, as well which vaccines for adults are recommended in the NIP. Guidelines from local sources tend to be more pertinent to primary care physicians, while medical professionals in specialized fields are more likely to refer to international guidelines. In order to provide a means of developing and updating guidelines, it might be necessary to establish multidisciplinary working groups focused on vaccination and other primary prevention strategies. These groups could create recommendations, guides, and consensus documents to improve awareness among HCPs and ensure that they are informed about the appropriate guidelines.

Most treatment protocols for immunocompromised patients do not include recommendations for vaccination and this is an area that needs to be addressed urgently. For example, no oncology treatment guidelines in Mexico mention vaccination as a preventive measure, other than human papillomavirus-related cancer. It is not desirable for individual specialties that treat immunocompromised patients to develop their own guidelines; rather, inter-society collaboration is needed to promote an integrative approach to vaccination in immunocompromised populations.

Improving adult vaccination rates, including vaccination against HZ, will require extended infrastructure and access. First, existing vaccination centers in hospitals and preventive medicine units should be strengthened, as it is more efficient to improve or expand established centers than it is to introduce new vaccinators. Later, training new vaccinators who focus on adults will be an important part of expanding access to vaccination, keeping in mind patients with debilitating conditions and immune-mediated diseases. Access can also be expanded by offering vaccination in pharmacies and shopping centers and other easy-to-reach venues. Such amenities are vital for non-vaccinating HCPs who need to refer patients for vaccination. These facilities will also improve health equity by bringing disease prevention closer to those who need it, not just the middle–high-income population.

## 5. Conclusions

Adult vaccination is not well established in Mexico, but situations like the COVID-19 pandemic have increased health and vaccination awareness among the public. We must take advantage of this moment to raise awareness about vaccination as a preventive measure at all stages of life and about all available vaccines for adults and older adults, as well as the concept of healthy aging among older adults. Although HZ does not have a high mortality rate, it is a very painful disease that can have devastating long-term complications and impact on quality of life. Improving vaccination rates against HZ in older adults and immunocompromised individuals in Mexico is of paramount importance. A number of barriers to vaccination exist and must be overcome. These include improving awareness of HZ and vaccine accessibility, developing and promoting guidelines and recommendations for vaccination, and expanding access to vaccination.

## Data Availability

Data sharing is not applicable to this article as no datasets were generated or analyzed during the current study.

## Trademark

Zostavax is a trademark of MSD. Shingrix is a trademark owned by or licensed to GSK. AS01 is a trademark owned by or licensed to the GSK group of companies.
